# Mercury exposure leading to functional vitamin B12 deficiency and subacute combined degeneration: a case report and literature review

**DOI:** 10.3389/ftox.2025.1580275

**Published:** 2025-09-09

**Authors:** Isidora Semnic, Valentino Rački, Olivia Perković, Vladimira Vuletić

**Affiliations:** ^1^ Department of Neurology, Clinical Hospital Centre Rijeka, Rijeka, Croatia; ^2^ Department of Neurology, Faculty of Medicine, University of Rijeka, Rijeka, Croatia; ^3^ Faculty of Medicine, University of Rijeka, Rijeka, Croatia

**Keywords:** mercury, intoxication, B12-deficiency, combined degeneration of spinal cord, extrapyramidal symptoms

## Abstract

**Introduction:**

The association between neurological symptomatology and heavy metal exposure has been reported in the literature. A few cases of extrapyramidal symptomatology and subacute combined degeneration have been described as manifestations of mercury intoxication. We highlight a case of a patient presenting with Parkinsonian features (tremor, rigidity, and bradykinesia), pyramidal deficits, dysarthria, paresthesia, mild cognitive decline, and emotional lability, with proven elevated mercury levels in blood and hair and elevated arsenic in urine.

**Case:**

A 60-year-old man, with history of mercury exposure while working at the Centre for Waste Management and Environmental Protection presented to a neurologist after 10 months of persistent tremors, muscle spasms, paresthesia, and irritability, followed by the onset of bradykinesia, slurred speech, rigidity, insomnia, and subtle cognitive decline. Laboratory investigations revealed functional vitamin B12 and vitamin D deficiencies, while toxicological quantitative analysis showed elevated blood mercury levels (15.2 μg/L) and hair root levels (3 μg/g). MRI of the brain was normal, whereas MRI of the posterior cervical spine detected signs of myelopathy. Florodeoxyglucose (FDG) Positron Emission Tomography (PET) of the brain revealed bilateral temporal and parietal glucose hypometabolism, most pronounced in the left inferior parietal and left superior temporal regions. Single-Photon Emission Computed Tomography (SPECT) imaging of dopaminergic neurons in the striatum was negative, and the patient was unresponsive to levodopa. Multivitamin therapy (vitamins B, E, and D) with selenium, in combination with symptomatic therapy (benzodiazepines, muscle relaxants, and antidepressants) provided minimal relief, leading to the introduction of N-acetyl cysteine, which resulted in moderate improvement of symptoms. Physical and speech therapy were of great importance in this case.

**Discussion:**

This case is unique because it represents the development of therapy-resistant extrapyramidal symptoms over 3 years of mercury exposure, likely leading to subacute combined degeneration due to functional vitamin B12 deficiency. Epidemiological data describe methylmercury poisoning, known as Minamata disease, which presents with -somatosensory deficits, ataxia, parkinsonism, dysarthria, and visual and hearing impairments.

**Conclusion:**

Toxicological screening for heavy metals in blood and urine should be considered in patients presenting with unexplained, levodopa-resistant extrapyramidal symptoms, behavioral and sleep disturbances, cognitive decline, and other non-specific neurological signs.

## 1 Case report

### 1.1 Introduction

Mercury exposure has been linked to various neurological deficits in the literature. Mercury is most commonly released into the environment through mining, industrial combustion, waste disposal, agriculture, medicine, and dental product management (Gençpınar et al., 2015). Different forms of mercury exhibit toxicity in the nervous, pulmonary, gastrointestinal, and renal systems (George et al., 2023). Human intoxication occurs mainly through occupational exposure via inhalation of elemental mercury vapors in industry settings, through consumption of seafood containing organic mercury (most commonly methylmercury), and via dermal contact with inorganic mercury compounds (such as mercuric chloride) (Yawei et al., 2021). Mercury is known to cause neuro-oxidative stress, mitochondrial dysfunction, and cellular damage (Basta et al., 2021). Public health disasters with endemic food poisoning happened in Minamata Bay, Japan, and Iraq (Balachandran et al., 2023). Classically described neurological signs, referred to as Minamata disease, include somatosensory deficits, ataxia, dystonia and parkinsonism (Ganguly and Jog, 2022), dysarthria, and visual and hearing impairments (Hong et al., 2012). In contrast to organic food poisoning, professional exposure is mainly due to elemental mercury exposure, which aligns with the risks in our patient’s work environment. Presentations of subacute and chronic mercury exposure include weakness, tremors, muscle spasms, dysarthria, and impaired cognitive skills (Gençpınar et al., 2015). Although acute intoxication typically results in elevated blood mercury levels, analysis of urine or hair samples can confirm chronic exposure, which correlates with more subtle symptoms such as peripheral neuropathy (Rebouças et al., 2024), irritability, and depression (Gençpınar et al., 2015). This report presents a patient who developed extrapyramidal, pyramidal, sensory, and cognitive symptoms, along with elevated mercury in blood and hair and a functional deficiency of vitamin B12, leading to a subacute combined degeneration.

### 1.2 Case presentation

**FIGURE 1:**
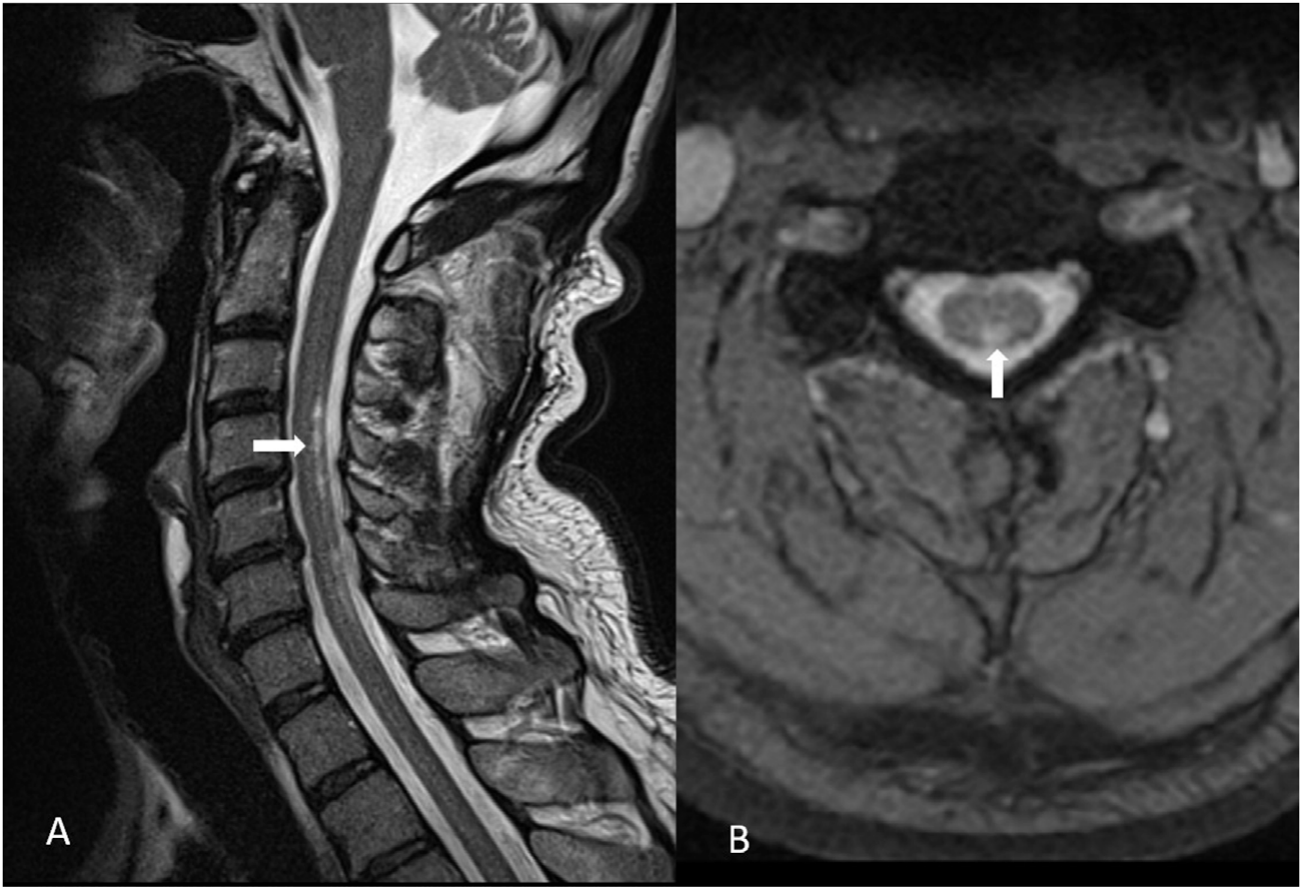
Morphological changes in the posterior cervical medullar region C3–C7 corresponding to a subacute combined degeneration (marked with arrows): **(A)** sagittal and **(B)** transversal plane.

A 60-year-old male patient was referred to our hospital with a 10-month history of gradually worsening tremors in his hands (predominantly in the fingers of the right hand), difficulties in fine motor activities, insomnia, irritability, and fatigue. He also reported muscle cramps at rest and tingling in his legs and feet. The patient had a history of treated hypertension and hyperlipidemia. His family history was negative for hereditary neurological and psychiatric diseases. He worked at a waste disposal Centre and reported frequent exposure to electrical devices, such as antennas, varnished packaging, and light bulbs. His social history and hobbies were unremarkable. The initial neurological status revealed positional and intentional tremor of the hands and slight instability. Initial extensive laboratory tests (complete blood count, kidney and liver tests, sodium, potassium, magnesium, TSH, free T4, T3, copper in serum and urine, ceruloplasmin, homocysteine, iron, folic acid, vitamin B12, vitamin D, and immunological tests) were within normal limits. Viral serology on EBV, B. *Borrelia*, HIV, and Hepatitis B and C was negative. Given his occupational exposure to heavy metals, toxicological analysis via atomic absorption spectrometry revealed elevated blood mercury levels (15.2 μg/L; reference range <5 μg/L), normal 24-h urinary mercury, slightly elevated blood arsenic (13.8 μg/L; RR < 10 μg/L), and significantly increased urinary arsenic (588.31 μg/L; RR < 25 μg/L), measured using inductively coupled plasma mass spectrometry. The mercury content of hair sample (3 cm long) was 3 μg/g, exceeding the standard reference range (RR < 1). Psychological testing showed an emotionally irritable profile, slightly disturbed working memory, and cognitive decline. Brain MRI was normal except for a small pineal cyst. Cervical spine MRI showed morphological changes (Figure 1). The results of the lumbar puncture included slight proteinorachia 704 mg/L (RR 170–370 mg/L), albuminorachia 471 mg/L (RR 144–336 mg/L), IGG concentration at 46 (RR 7,4–39 mg/L), and reduced function of the blood–CSF barrier (96; RR > 130) without other abnormalities. Low levels of Hg were detected in cerebrospinal fluid, though accurate quantification was not possible. Neurodegenerative markers (total tau, amyloid beta 40/42, and phosphorylated tau 181) were negative. During the workup, he experienced a decline in motor and cognitive functions, including increased muscle stiffness and tremor, spastic and rigid gait, paresthesia, hypoesthesia, and reduced proprioception. Further laboratory findings revealed a functional vitamin B12 deficiency due to elevated methylmalonic acid, and parenteral vitamin B12 therapy was initiated, along with pregabalin, cholecalciferol, vitamin E, and selenium. N-acetylcysteine was initiated with a daily dose of 4200 mg, along with baclofen. Dopaminergic therapy with levodopa/benserazide and levodopa/carbidopa were trialed on two occasions, without improvement to the extrapyramidal symptomatology. The therapy caused a stabilization of symptoms. In order to follow the trend of mercury in the body, the analysis was repeated 10 months after the initial test at another laboratory and confirmed elevated blood mercury levels at 18,84 μg/L (RR < 5 μg/L) and in the hair root 1,37 mcg/g (RR less than 0,6), and a diagnosis of mercury poisoning secondary to professional waste exposure was carried out. Over the past 3 years, repeated MRI scans of the brain and cervical spinal cord remained unchanged. Dopaminergic SPECT imaging revealed no signs of reduced dopamine transporter availability in the region of the corpus striatum. FDG-PET of the brain demonstrated bilateral temporal and parietal hypometabolism, most pronounced in the right and left inferior parietal regions, the left superior temporal region, and the Broca region. He exercised regularly and started swimming with positive effects. Our patient reports improvement in general condition and daily activities but remains moderately neurologically impaired (spastic dysarthria, asymmetrical tetraparesis—more dominant weakness and hyperreflexia in the right hand and leg, akinetic tremor of the right hand, postural tremor of all extremities, dysmetria, hypoesthesia, and generally diminished vibration sensibility). Timeline review is presented in the Table 1.

**TABLE 1:** Patient report.

Category	Details
Demographics	Male, 60 years old
Chief complaint	10-month history of progressive hand tremors (right dominant), fine motor difficulties, insomnia, irritability, and fatigue
Additional symptoms	Muscle cramps at rest and tingling in legs and feet
Medical history	Hypertension and hyperlipidemia (both treated)
Family history	Negative for neurological or psychiatric conditions
Occupational exposure	Worked at a waste disposal center; frequent contact with electrical devices (antennas, varnished packaging, and light bulbs)
Social and lifestyle history	Unremarkable; no notable hobbies or substance use
Initial neurological exam	Positional and intentional hand tremor, slight instability
Laboratory findings (initial)	Normal: CBC, kidney/liver function, electrolytes, thyroid panel, copper (serum/urine), ceruloplasmin, homocysteine, iron, folate, vitamin B12, vitamin D, and immunology. Viral serologies (EBV, *Borrelia*, HIV, HBV, and HCV): negative
Toxicology results	**Blood mercury**:^a^ 15.2 μg/L (↑, RR < 5); **urine mercury**:^b^ normal; **blood arsenic**:^c^ 13.8 μg/L (↑, RR < 10); **urine arsenic**:^d^ 588.31 μg/L (↑, RR < 25); and **hair mercury**:^e^ 3 μg/g (↑, RR < 1)
Psychological Findings	Emotional irritability, mild cognitive decline, and working memory deficits
Imaging	**Brain MRI**:^f^ normal except for small pineal cyst; **cervical MRI**:^f^ posterior cord changes (C3–C7); **SPECT**:^g^ no dopaminergic loss; and **FDG-PET**:^h^ bilateral parieto-temporal hypometabolism
CSF analysis	Slight proteinorachia (704 mg/L, ↑); albuminorachia (471 mg/L, ↑); elevated IgG (46 mg/L, ↑); blood–brain barrier dysfunction (96, ↓); neurodegenerative markers: negative
Functional deficiency	Functional vitamin B12 deficiency (elevated methylmalonic acid)
Treatment initiated	Parenteral vitamin B12 **supplements**:^i^ pregabalin, cholecalciferol, vitamin E, and selenium; **N-acetylcysteine**:^j^ 4200 mg/day; **baclofen** ^k^- and **levodopa-based therapy**:^l^ no effect on symptoms
Clinical course	Progressive motor and cognitive decline, with spasticity, rigidity, paresthesia, hypoesthesia, and reduced proprioception
Follow-up toxicology (10 months)	**Blood mercury**:^a^ 18.84 μg/L (↑, RR < 5) and **hair mercury**:^e^ 1.37 μg/g (↑, RR < 0.6)
Final diagnosis	Mercury poisoning secondary to occupational exposure
Recent imaging	Brain/cervical MRI: Stable over 3 years
Rehabilitation	Regular exercise and swimming with subjective improvement
Current neurological status	Moderate impairment: spastic dysarthria, asymmetrical tetraparesis (R > L), right-sided akinetic tremor, postural tremor (all limbs), dysmetria, hypoesthesia, and reduced vibration sense

^a^ Concetration of mercury in the blood.

^b^ Concetration of mercury in the urine.

^c^ Concetration of arsenic in the blood.

^d^ Concetration of arsenic in the urine.

^e^ Concetration of mercury in the hair.

^f^ Magnetic Resonance Imaging.

^g^ Single-Photon Emission Computed Tomography.

^h^ Florodeoxyglucose-Positron Emission Tomography.

^i^ Concentrated pills, powders or other forms that contain vitamins, meant to supplement the diet when it doesn’t provide enough of certain vitamins for normal body functions.

^j^ An acetylated form of the amino acid L-cysteine and a powerful antioxidant.

^k^ Medication used to treat muscle spasticity.

^l^ The most effective treatment for the motor symptoms of Parkinson’s disease.

## 2 Discussion

We present a neurological case of mercury intoxication, likely resulting from occupational exposure during the management of electrical waste. The possibility of mercury poisoning should be considered in patients presenting with unexplained tremor, motor symptoms, paresthesia, insomnia, depression, or other sleep disturbances (Wang and Fang, 2021). Our understanding of mercury’s neurotoxic effects stems from environmental poisoning episodes that occurred in Minamata Bay and Yatsushiro Sea, Japan (1950–1968) (Yorifuji et al., 2023), and Iraq (1971–1972) (Bakir et al., 1973) through the consumption of contaminated fish and seafood (Rebouças et al., 2024). Hunter–Russell triad was described in severe cases of organic mercury intoxication (visual field constriction, hearing deficit, ataxia, and sensory disorders) (Shimohata et al., 2015). Milder forms included sensory neuropathy and psychomotor changes (Takaoka et al., 2023). Notably, mercury vapor accumulates in the brain in higher quantities than equivalent doses from oral or parenteral exposure due to rapid uptake following inhalation (O'Donoghue et al., 2020). Additionally, mercury vapor rapidly crosses the blood–brain barrier, oxidizes to Hg^2+^ in brain tissue, and remains there for years (Bjørklund G et al., 2017). Elemental mercury exposure was associated with the industrial environment in which the patient worked.

Upon entering the central nervous system, mercury undergoes demethylation, and mercuric ions accumulate in the nervous tissue, interacting with different amino-acid groups, leading to mitochondrial damage, increased excitatory amino acid release, proteomic and oxidative stress, and cellular destruction (Basta et al., 2021). Pyramidal cells, astrocytes, Purkinje cells, and deep cerebellar nuclei are classically involved (Syversen and Kaur, 2012). Peripheral nervous system involvement is more common with elemental and inorganic mercury intoxication (Jackson, 2018). Chronic elemental mercury exposure is characterized by tremor, sleep and memory disturbance, emotional changes, headache, peripheral neuropathy, and ataxia (Jackson, 2018), and notably, it may mimic parkinsonism, depression, and Alzheimer’s disease (Olson, 2024). At low levels of mercury, patients have nonspecific symptoms such as headache and nausea, but at higher levels, ataxia, encephalopathy, and a neuropsychiatric syndrome known as erethism (excessive anxiety and irritability) are described (Jackson, 2018).

We have described a case of a man exposed to inorganic mercury in a contaminated industrial environment, presenting with motor and psychiatric disorders that altogether significantly altered and limited his daily life. He did not have cerebellar or visual problems as in previously described cases. A recently described case report by Kuo et al. (2024) highlighted the likely connection between mercury exposure and the development of secondary parkinsonism, while the combined damage to the extrapyramidal, pyramidal, and sensory functions is likely due to the subacute combined degeneration and functional B12 deficiency. Moreover, studies support the connection between hair mercury levels and reduced cognitive functions (Santos-Lima et al., 2020): one study found that mercury concentrations in hair higher than 10.0 μg/g correlate with lower results in cognitive and semantic verbal fluency tests (Reuben et al., 2020; Oliveira et al., 2021). The exposure level in our patient was lower than that reported in the literature but appeared sufficient to cause a measurable decline in verbal fluency and executive function.

Considering mercury exposure to other vitamin deficiencies, it has been shown that heavy metals, such as lead, mercury, and cadmium, can impede vitamin D metabolism by affecting key enzymes of the vitamin D synthesis, such as 1α-hydroxylase and 24-hydroxylase (Gu et al., 2024), which are crucial for converting inactive vitamin D into its active form. Furthermore, methylmercury influences the activity of matrix metalloproteinase-9 (MMP-9), which contributes to the breakdown of the cytoskeleton and weakens the connections between cells (Khan et al., 2017).

Mercury induces significant disruption of selenium homeostasis, not only through sequestration via the formation of inert Hg–Se complexes but also by inhibiting the transmembrane trafficking of selenium across tissues. These combined effects imply that selenium repletion following mercury exposure may be pharmacologically limited or biologically ineffective (Dolgova et al., 2019). Disruption of selenoenzyme function within the cellular redox system leads to an accumulation of intracellular reactive oxygen species (ROS), ultimately resulting in mitochondrial dysfunction (Moniruzzaman et al., 2021). Animal research showed that mercury exposure markedly decreased ascorbic acid levels in the brain, adrenal glands, and spleen of experimental animals. As a key endogenous antioxidant, vitamin C is frequently depleted under conditions of mercury-induced oxidative stress, reflecting its role in counteracting ROS (Moniruzzaman et al., 2021). In one study, serum antioxidants, such as vitamin A and E, were found to be lower in dental workers exposed to mercury than those in a control group. The possible mechanism involves the interaction of mercury with long-chain conjugated double bonds present in the vitamins, rendering them susceptible to damage by various reactive oxygen species (Mohsen et al., 2013).

Interestingly, to the best of our knowledge, mercury has not previously been linked with subacute combined degeneration. Mercury has formerly been described in combination with megaloblastic anemia—a hallmark of vitamin B12 deficiency—but this was not observed in our patient (Chang et al., 2006). Current findings on the metabolic links between mercury and vitamin B12 involve competitive enzymatic reactions as mercury inhibits methionine synthase, a key enzyme that regenerates methionine from homocysteine (Gallagher and Meliker, 2011). A cross-sectional study in children revealed that mercury correlated with higher methylmalonic acid (Gallagher and Meliker, 2011), as was the case in our study. Mercury likely impacts the methylation of vitamin B12 (Imura et al., 1971; Chemaly, 2002), thus possibly leading to functional deficits despite normal serum levels, often accompanied by increased methylmalonic acid levels. Subacute combined degeneration is known to be caused even in cases of functional B12 deficiency (Hara et al., 2020) and is a plausible cause in our patient, with a metabolic link to increased mercury levels. We administered N-acetylcysteine to our patient for 1 month while maintaining a regular regimen of multivitamin antioxidants, including vitamins B12, D, and E and selenium. N-acetylcysteine aids in mercury detoxification by enhancing renal mercury excretion through its chelating properties, by reducing oxidative stress, and by promoting glutathione synthesis (Schwalfenberg, 2021). Additionally, N-acetylcysteine mitigates neurotoxic effects by combating oxidative stress in *in vivo* animal models, which is a key mechanism of mercury-induced cellular damage (Falluel-Morel et al., 2012). Its ability to replenish intracellular glutathione reserves further contributes to cellular protection against heavy metal toxicity (Schmitt et al., 2015). Selenium redistributes mercury from the brain into the blood and protects against mitochondrial damage (Spiller et al., 2021). Vitamin E is one of the most effective antioxidants for preventing lipid peroxidation (Niki, 2015), and experimental studies have shown that it reduces mercury toxicity (GS et al., 2012).

### 2.1 Patient perspective

“My problems, including muscle cramps, tremor, gait difficulty, and anxiety, started five years ago. Laboratory, toxicological, and neuroradiological investigations were conducted, and I have been regularly followed by a neurologist with the support of psychotherapy, speech therapy, and physical treatment. A year ago, my health condition became stable, and I feel better. We should all react promptly if there is a suspicion of the potential negative effects of toxic substances in our environment. I am thankful to the team of physicians who helped me.”

## 3 Conclusion

This case highlights the importance of considering mercury poisoning in patients presenting with unexplained levodopa-resistant parkinsonism, motor deficits, paresthesia, cognitive decline, and other non-specific neurological and psychiatric symptoms, particularly in individuals with known occupational exposure to heavy metals. Our findings suggest a possible novel association between chronic mercury exposure and subacute combined degeneration due to functional vitamin B12 deficiency. This underscores the significance of conducting thorough toxicological screening, including blood, urine, and hair analyses. Multimodal therapeutic management, incorporating N-acetylcysteine, multivitamin antioxidant therapy, and physical and speech rehabilitation, played a critical role in stabilizing and moderately improving the patient’s condition. Further studies are warranted to explore the mechanistic links between mercury, oxidative stress, and vitamin B12 metabolism and better understand the long-term outcomes of such complex presentations.

## Data Availability

The original contributions presented in the study are included in the article/supplementary material; further inquiries can be directed to the corresponding author.
